# Association Between Health-Related Behaviors and Health Status and Hydration Status in Polish Adults

**DOI:** 10.3390/nu17162597

**Published:** 2025-08-09

**Authors:** Joanna Frąckiewicz, Kacper Szewczyk

**Affiliations:** Department of Human Nutrition, Institute of Human Nutrition Sciences, Warsaw University of Life Sciences (WULS-SGGW), 02-787 Warsaw, Poland; kacper_szewczyk@sggw.edu.pl

**Keywords:** health-related behaviors, health status, hydration status, consumption frequency, beverages, anthropometric measurements, urine, adults

## Abstract

**Objectives:** The health of the adult population is the result of many interacting variables, with health behaviors and lifestyle playing a key role. This study aimed to identify associations among health-related behaviors and health and hydration status in Polish adults. **Methods:** The completion of a beverage frequency questionnaire (FFQ) was undertaken by a total of 337 participants. Blood pressure (BP), anthropometric parameters, and body composition were measured. Urine samples were analyzed for specific gravity (USG), osmolality (Uosm), and potential hydrogen value (pH). Health-related behaviors were assessed using the Health Index Score (HIS), classifying participants into two groups: unhealthy habits (0–2 HIS group) and healthy habits (3–5 HIS group). Odds ratios (ORs) and 95% confidence intervals (CIs) were calculated. **Results:** Approximately 30% of participants (*n* = 115) exhibited unhealthy habits. Individuals in the 0–2 HIS group were more likely to be male, smoke, have low physical activity, be overweight or obese, sleep < 6 h, and/or consume alcohol ≥ 2 times/week. In contrast, higher HIS (3–5) was associated with female gender, non-smoking, moderate-to-high physical activity, normal body mass index (BMI), adequate sleep, and consuming alcohol < 2 times/week. Fatigue during the day (OR: 1.45), waist circumference (WC) (OR: 1.35), and Uosm (OR: 1.87) increased un-healthy habits. Conversely, greater consumption of non-carbonated mineral water (OR: 0.54) was linked to lower unhealthy habits. **Conclusions:** The HIS and hydration-related parameters can complement the assessment of the health status of the adult population and the identification of groups requiring special support in health promotion interventions.

## 1. Introduction

The health of the adult population is the result of many interacting variables, with health behaviors and lifestyle playing a key role [1]. Contemporary epidemiological and clinical studies emphasize that physical activity, weight control, avoiding stimulants such as tobacco and excessive alcohol consumption, and getting enough sleep are fundamental to preventing many chronic diseases, including cardiovascular, metabolic, and cancer diseases [2]. Incorrect health habits can lead to obesity, hypertension, insulin resistance, and metabolic disorders, significantly reducing quality of life and prolonging periods of disability [3,4].

In scientific research, the assessment of health status requires the use of objective indicators that allow for precise analysis of physiological and metabolic body functions. The most commonly used indicators include blood pressure, a direct marker of cardiovascular disease risk, and handgrip strength, an indicator of general muscle strength and physical fitness associated with hospitalization and mortality risk [5,6]. Waist circumference and body composition (e.g., percentage of body fat) enable the distribution of body fat to be assessed, which is important in terms of metabolic and cardiovascular risk [7,8].

Although often underestimated, an equally important aspect of health is the body’s hydration status [9]. Proper hydration is essential for maintaining homeostasis, regulating osmotic pressure and transporting nutrients, as well as ensuring the proper functioning of the nervous system and kidneys [10]. Objective indicators such as urine specific gravity and osmolality are used in scientific studies to allow for precise assessment of hydration status [11]. Analyzing the frequency of beverage consumption provides additional information on hydration habits, which is important for preventing dehydration, particularly among adults leading an active lifestyle [12].

Previous scientific literature has often focused on individual lifestyle elements or single health status aspects, without taking the simultaneous and comprehensive analysis of many health behaviors and their impact on physiological and metabolic parameters into account. Furthermore, the relationship between health behaviors and hydration status is not well understood, indicating the need for further research in this area [13].

Composite indicators are essential in population studies for comprehensive health assessment because they allow for the integration of many aspects of bodily functioning into a single numerical value. This facilitates the analysis of the relationship between lifestyle and physiological health and allows for comparisons between groups [14]. In this study, we used the Health Index Score (HIS), which takes into account five diverse yet key health behaviors: physical activity (physical activity factor), body mass index (BMI) (metabolic factor), smoking and alcohol consumption (behavioral risk factors), and sleep (regenerative factor). This index reflects the multidimensional nature of lifestyle and allows for a more comprehensive assessment of its links to traditional health indicators and hydration markers [13]. Although the HIS was originally developed as an overall health indicator, its components may also influence the body’s water and electrolyte balance. For example, physical activity affects daily fluid requirements and increases water loss through sweating [15], while BMI—reflecting the ratio of fat to lean mass—is associated with total body water [16]. By integrating five key health-related behaviors, the HIS provides a comprehensive lifestyle assessment that may be related to hydration markers such as urine specific gravity, osmolality, and beverage consumption. This offers new insights into the relationship between daily habits and water homeostasis; however, further research is needed to better understand these associations.

This study, therefore, aims to comprehensively assess the relationship between key health behaviors—physical activity, BMI, smoking, alcohol consumption, and hours of sleep—and health status, as measured by parameters such as blood pressure, handgrip strength, waist circumference, and body composition. Additionally, the study includes an assessment of body hydration status using objective measures of specific gravity (USG) and urine osmolality (Uosm), as well as an analysis of beverage consumption frequency. This approach will improve our understanding of how health behaviors affect the functioning of the adult body, which could have significant implications for clinical practice and preventive measures.

## 2. Materials and Methods

### 2.1. Study Design and Participants

The principles of the Declaration of Helsinki were followed in this observational study, which was conducted between May 2017 and February 2020. Ethical approval was granted by the Ethics Committee of the Faculty of Human Nutrition and Consumer Sciences at the Warsaw University of Life Sciences, Warsaw, Poland, on 11 April 2017 (Resolution No. 05p/2017). Written informed consent was obtained from each participant, constituting a prerequisite for inclusion in the study.

The convenience-sampled, cross-sectional study was designed to evaluate the general adult population. Participants were recruited from a variety of social backgrounds, covering both urban and rural areas, and representing a wide range of economic statuses and educational levels. A total of 450 people, ranging in age from 17 to 66, were included in the initial data collection; however, only participants aged 18 to 40 years were included in the final analyses to ensure a homogeneous study group. Individuals under the age of 18 and over 40 were excluded due to their lower representation and a higher likelihood of not meeting the inclusion criteria.

**Figure 1:**
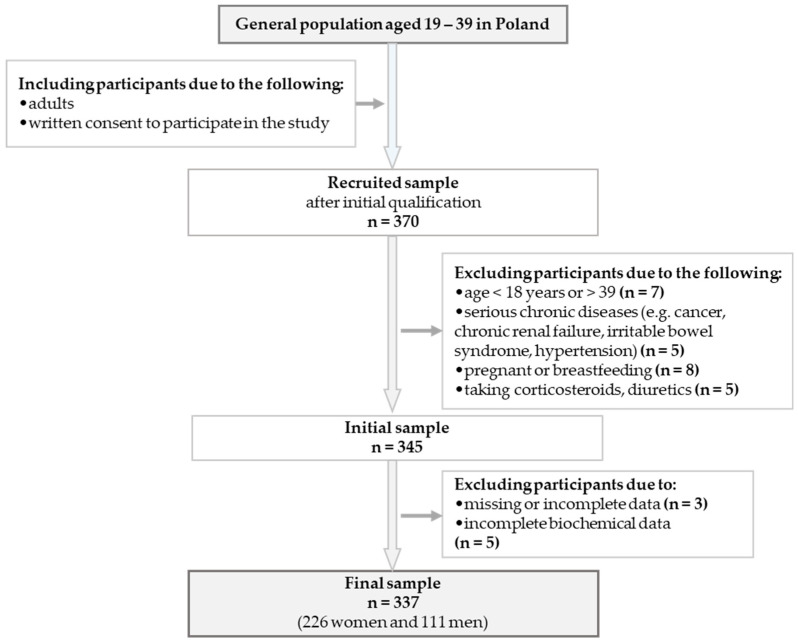
Flowchart showing the study design and data collection process.

A total of 337 participants (226 women and 111 men) were ultimately included in the analysis. Inclusion criteria required participants to be in the specified age range and capable of providing written informed consent. Exclusion criteria encompassed the inability to provide informed consent, diagnosed acute or chronic renal failure, cancer, hypertension, or irritable bowel syndrome. Those taking corticosteroids, diuretics, or other medications that could affect the results of the study were also excluded. Additional exclusion criteria included recent (within three days) episodes of vomiting, diarrhea, or fever, as well as being permanently bedridden or reliant on a wheelchair. The study excluded pregnant or breastfeeding individuals, as well as those with incomplete or missing data. Furthermore, participants with endoprostheses, pacemakers, stents, or metallic sutures in their heart or blood vessels were not eligible to participate due to the use of bioelectrical impedance analysis (BIA). Figure 1 shows the process of recruiting and retaining participants.

### 2.2. Sociodemographic Data

A survey method was employed to collect general sociodemographic information from the participants. The questionnaires were completed by the respondents in the presence of a trained interviewer to ensure clarity and consistency in responses. Participants were asked to provide data on the following variables: age (continuous variable), gender (male or female), educational level (categorized as secondary/‘I study’ or higher), and place of residence (village, town, or city). Additionally, self-reported data were collected on economic status (very poor/poor or average/very good) and health status (very poor/poor or average/very good).

### 2.3. Beverage Consumption Data

Information about diet was collected using a Food Frequency Questionnaire (FFQ). The relative validity of the FFQ was evaluated in a subgroup of 60 Polish participants aged 20–30 years from the study area by comparison with two repeated 4-day dietary records [17]. The validation results indicate moderate to high agreement between the FFQ and the reference method (24 h dietary recalls), with beverage correlations of 0.36–0.9 and acceptable limits of agreement. This information is provided to increase the transparency and credibility of the measurement tool [17]. The FFQ was completed under the supervision of an experienced dietitian to ensure procedural standardization and minimize the risk of errors in data collection. The direct involvement of the dietitian also provided essential support, allowing for the clarification of any ambiguities and enhancing the accuracy of the responses. The FFQ collected detailed information on the frequency of consumption of a broad range of beverages, including both non-alcoholic and alcoholic drinks. The beverages assessed were tea, coffee, herbal infusions, milk, fermented milk drinks (both plain and flavored), mineral water (carbonated and non-carbonated), juices (fruit, vegetable, and mixed), non-carbonated fruit drinks, sweetened carbonated drinks, tea-based beverages, cola drinks, energy drinks, isotonic drinks, non-alcoholic beer, beer, wine, vodka, and alcoholic drinks. Beverage consumption frequency was categorized as follows: 0—never; 1—less than 1 serving per month; 2—1–3 servings per month; 3—1–2 servings per week; 4—3–4 servings per week; 5—5 servings per week; 6—1 serving per day; and 7—2 or more servings per day.

### 2.4. Health Status

#### 2.4.1. Blood Pressure

Systolic and diastolic blood pressure (SBP and DBP) measurements were conducted using a standard automatic sphygmomanometer, the SureSigns VM6 Cardiac Monitor (Philips Medical Systems, Andover, MA, USA). Prior to measurement, participants were instructed to abstain from smoking or the use of stimulants and to ensure bladder voiding. Measurements were performed following a 10 min rest period. Participants were seated in a relaxed, upright position with feet flat on the floor and without crossing their legs. The upper limb was supported on a table at heart level, and the cuff was carefully selected to match the circumference of the upper arm, positioned on its middle third. Blood pressure was measured in the morning between 7:00 and 9:00 a.m. Two measurements were taken during the same visit at intervals of 1–2 min. If the difference between the first and second readings exceeded 10 mmHg, a third measurement was obtained [18].

Standard reference values for blood pressure were defined as follows: SBP < 130 mmHg and DBP < 85 mmHg, regardless of gender [19,20].

#### 2.4.2. Anthropometric Measurements

The anthropometric measurements collected in the study included body weight (BW), height (H), waist circumference (WC), hip circumference (HC), and handgrip strength (HGS). These measurements were performed in duplicate following standardized protocols and under the supervision of trained researchers to ensure consistency and reliability [21]. Participants were instructed to wear minimal clothing and to remove their shoes prior to measurement. Body weight was measured to the nearest 0.1 kg using an electronic digital scale (SECA 799, Hamburg, Germany), and height was assessed with a stadiometer (SECA 220, Hamburg, Germany), recorded to the nearest 0.1 cm. BMI was subsequently calculated and classified according to the World Health Organization (WHO) standards for adults: BMI < 18.5 kg/m^2^ was classified as underweight, 18.5–24.9 kg/m^2^ as normal weight, 25.0–29.9 kg/m^2^ as overweight, and ≥30.0 kg/m^2^ as obese [22]. For the purposes of statistical analysis, overweight and obese individuals were grouped into a single category.

WC and HC were measured using a non-stretchable, tensile-resistant measuring tape (SECA 201, Hamburg, Germany), ensuring uniform application of pressure during the measurement.

HGS was assessed using a hydraulic hand dynamometer (SAEHAN Corporation, Masan, Changwon, Republic of Korea) and recorded to the nearest 0.5 kg. Measurements were taken for both the dominant and non-dominant hand, with participants in a standing position and exerting maximum effort. A rest period of approximately two minutes was observed between each measurement [23]. To enhance accuracy and reduce variability, the mean values for each parameter were calculated from the repeated measurements [21].

#### 2.4.3. Body Composition

Participants underwent bioelectrical impedance analysis (BIA) assessments while wearing light clothing and having removed all metal objects to prevent measurement interference. Prior to testing, participants received detailed instructions regarding appropriate pre-measurement preparation. This included fasting or abstaining from food for at least 4 h prior to measurement, avoiding coffee for at least 4 h, refraining from alcohol consumption for 48 h, and avoiding physical activity for a minimum of 12–24 h before the procedure [24]. Each participant was measured twice on the same day to ensure consistency. During the assessment, participants were in a supine position, and two self-adhesive, disposable skin electrodes (Maltron International Ltd., Rayleigh, UK) were applied to specific anatomical locations on the right hand and foot. On the hand, the red electrode was positioned on the dorsal side at the base of the second and third fingers, while the black electrode was placed on the wrist in a straight line below the first electrode. On the foot, the red electrode was placed on the dorsal side at the base of the second and third toes, and the black electrode was affixed just above the ankle.

The Maltron BioScan 920 (Maltron International Ltd., Rayleigh, UK) operates using a multi-frequency bioimpedance current of 400 µA at frequencies of 5 kHz, 50 kHz, 100 kHz, and 200 kHz. Previous studies have demonstrated that total body water (TBW) measurements obtained via BIA correlate well with conventional hydration biomarkers and fluid balance indicators, supporting its validity and reliability in hydration and body composition research [25,26,27].

### 2.5. Hydration Status

#### Biochemical Analysis in Urine

To reduce the chance of deliberate overhydration before the assessment, the participants were told not to change their usual dietary and fluid intake habits. This approach was designed to provide a natural and accurate assessment of their typical hydration levels. Furthermore, prior to urine collection, participants were given the same dietary recommendations as for the body composition analysis. Participants performed the necessary intimate hygiene procedures before collecting their first morning urine sample, which was obtained as soon as they woke up. A midstream urine sample (50–100 mL) was collected in a sterile, disposable urine container. All samples were anonymized using coded identifiers and stored at a temperature of approximately 4–8 °C for no longer than one hour. Subsequently, samples were transferred to −20 °C storage conditions for up to one month to preserve sample stability prior to analysis. Urine samples were analyzed for USG, potential hydrogen value (pH), and Uosm. The assessment of USG and pH was conducted in a certified laboratory using standardized and validated methods, including refractometry for USG, and within reference ranges commonly accepted in the scientific literature. The reference range for USG was 1.010 to 1.030 g/cm^3^, while acceptable pH values ranged from 5.0 to 7.5. Uosm was measured using a freezing point osmometer (cryoscopy method) (Marcel OS3000 osmometer, Warsaw, Poland). The osmometer was calibrated prior to each use to ensure precise readings. According to widely accepted hydration benchmarks, Uosm values exceeding 700 mOsm/kg are indicative of mild dehydration, while values above 800 mOsm/kg signify dehydration. In contrast, Uosm values below 700 mOsm/kg are considered indicative of adequate hydration status [28,29].

### 2.6. Health-Related Behaviors and Health Index Scores (HISs)

Health-related behaviors assessed in this study included physical activity, BMI, smoking, alcohol consumption, and sleep duration. Physical activity levels were evaluated using the International Physical Activity Questionnaire (IPAQ), and responses were categorized into a binary variable based on whether participants met the physical activity guidelines. The IPAQ categorizes activity levels as below 600 (low), 600–1500 (moderate), or above 1500 (high) metabolic equivalent of work (MET) min/week [30]. Participants were classified as having a normal BMI (<25.0 kg/m^2^) or as overweight/obese (≥25.0 kg/m^2^). Smoking status was categorized dichotomously as smoker or non-smoker. Alcohol consumption was assessed based on frequency, with participants classified as light drinkers (<2 times/week) or heavy drinkers (>2 times/week). Sleep status was recorded based on self-reported average nightly sleep duration and dichotomized into <6 h or ≥6 h of sleep per night [13].

Each of the five health-related behaviors was recoded into binary variables: healthy behaviors were coded as 1 and unhealthy behaviors as 0. A composite health behavior index was then calculated by summing the scores of the five recoded behaviors, resulting in a total score ranging from 0 to 5. For analytical purposes, HISs of 3, 4, or 5 were categorized as indicative of healthy habits. Conversely, scores ranging from 0 to 2 were defined as indicative of unhealthy habits [13].

### 2.7. Statistical Analysis

All statistical analyses were carried out using STATISTICA version 13.3 (TIBCO Software Inc., StatSoft, Palo Alto, CA, USA), with a significance level of α = 0.05. The a priori sample size was estimated using G*Power software version 3.1.9.7, assuming a medium effect size (Cohen’s *d* = 0.5), a two-tailed Wilcoxon–Mann–Whitney test, an alpha level of 0.05, and a statistical power of 0.80. The minimum required sample size was determined to be 64 participants per group (total N = 128). The normality of data distributions was assessed by the Shapiro–Wilk test. Results are presented as median and range, quartile 1 (Q1) to quartile 3 (Q3) for continuous variables, or as percentages (%) for categorical variables. Participants were categorized into study groups according to the number of HIS components, stratified into two groups: 0–2 HIS and 3–5 HIS. Group differences were evaluated using Pearson’s chi-squared test for categorical variables and the Mann–Whitney U test for continuous variables. The partial correlation between the chosen variables for health status and hydration status, and the number of HIS components was explored using the Spearman correlation test. Furthermore, a multivariate logistic regression analysis, adjusted for gender, age, and place of residence (Model 1) and adjusted for gender, age, place of residence, education, and economic status (Model 2), was conducted to identify factors associated with the 0–2 HIS group (indicative of unhealthy habits). Odds ratios (ORs) with corresponding 95% confidence intervals (CIs) were calculated, and the statistical significance of these ORs was assessed using Wald’s test. Finally, a post hoc power analysis was conducted using G*Power to confirm the adequacy of the achieved sample size. Assuming a medium effect size (Cohen’s *d* = 0.5), α = 0.05 (two-tailed), and sample sizes of *n* = 115 and *n* = 222, the achieved power was 0.991, indicating a very high probability of detecting a true effect.

## 3. Results

The following order has been used to present the findings in this section: the sociodemographic characteristics (Table 1), beverage consumption patterns (Table 2), anthropometric data (Table 3), correlations between key variables (Table 4), and the results of multiple regression analysis (Table 5).

### 3.1. Characteristics of the Study Group

The final sample comprised 337 participants, of whom 66% are women. Table 1 presents a comparison of baseline characteristics between two groups stratified by HIS: 0–2 HIS group (*n* = 115) and 3–5 HIS group (*n* = 222). Mean age is similar in both groups (24.8 ± 4.9 for 0–2 HIS vs. 23.7 ± 4.2 for 3–5 HIS). Secondary education or current study was declared by the majority of respondents (69.3%), a city was declared as a place of residence by 58.7% of respondents, average or very good financial status was declared by 72.0% of respondents, and good or very good health was declared by 77.0% of respondents. These variables did not show a significant difference between the two HIS groups.

**Table 1:** Study population characteristics by HIS groups.

Variables	0–2 HIS (*n* = 115)		3–5 HIS (*n* = 222)		*p*-Value
	*n*	%	*n*	%	
Age (years)	23.0 [22.0–25.0] *		23.0 [21.0–25.0] *		0.111
Gender					
Female	64	55.6	162	72.9	0.001
Male	51	44.4	60	23.1	
Education					
Secondary/‘I study’	80	69.6	153	68.9	0.903
Higher	35	30.4	69	31.1	
Place of residence					
Village	19	16.5	45	19.4	0.368
Town	30	26.1	44	20.6	
City	66	57.4	133	60.0	
Economic status					
Very poor/poor	35	30.4	57	25.7	0.352
Average/very good	80	69.6	165	74.3	
Health status					
Very poor/poor	27	23.5	50	22.5	0.843
Average/very good	88	76.5	172	77.5	
Cigarette smoking					
Yes	34	29.6	4	1.8	0.001
No	81	70.4	218	98.2	
Physical activity					
No/low	61	53.0	37	16.7	0.001
Moderate	34	29.6	121	54.5	
High	20	17.4	64	28.8	
BMI					
<18.5 (kg/m^2^)	3	2.6	19	8.5	0.001
18.5–24.9 (kg/m^2^)	51	44.4	186	83.8	
≥25.0 (kg/m^2^)	61	53.0	17	7.7	
Sleeping status					
<6 h	90	78.3	12	5.4	0.001
>6 h	25	21.7	210	94.6	
Alcoholic drinking status					
<2 times/week	24	20.9	207	93.2	0.001
>2 times/week	91	79.1	15	6.9	

* Median [Q1–Q3]; HIS, Health Index Score; BMI, body mass index.

A significantly higher percentage of individuals who smoked were in the 0–2 HIS group (29.6%) compared to the 3–5 HIS group (1.8%). Moderate (54.5%) and high physical (28.8%) activity levels are significantly more prevalent in the 3–5 HIS group. Overweight/obesity (53.0%) is significantly more prevalent in the lower HIS group (0–2 HIS), while normal weight (83.8%) is more prevalent in the higher HIS group (3–5 HIS). A significantly higher percentage of individuals sleeping more than 6 h are in the 3–5 HIS group (94.6%) compared to the 0–2 HIS group (21.7%). A significantly higher percentage of individuals drinking ≥2 times/week are in the 0–2 HIS group (79.1%) compared to the 3–5 HIS group (6.9%).

### 3.2. Frequency of Beverage Consumption

Table 2 compares the frequency of consumption of selected beverages by two groups of participants divided on the basis of HIS. The table shows significant differences in the frequency of beverage consumption between subjects in the higher and lower HIS groups. Participants in the 3–5 HIS group significantly more often consumed natural fermented milk drinks and non-carbonated mineral water, and significantly less often reached for flavored fermented milk drinks, non-carbonated fruit drinks, sweetened carbonated drinks, tea drinks, energy drinks, beer, and vodka.

**Table 2:** Frequency of consumption of selected beverages by HIS groups.

Variables	0–2 HIS (*n* = 115)	3–5 HIS (*n* = 222)	*p*-Value
	Median [Q1–Q3]		
Tea	6.0 [4.0–7.0]	6.0 [4.0–7.0]	0.668
Coffee	5.0 [2.0–7.0]	5.0 [2.0–6.0]	0.232
Herbal infusions	2.0 [1.0–4.0]	2.0 [1.0–4.0]	0.548
Milk	3.0 [2.0–6.0]	4.0 [2.0–6.0]	0.878
Natural fermented milk drinks	2.0 [1.0–4.0]	3.0 [2.0–4.0]	0.048
Flavored fermented milk drinks	3.0 [1.0–4.0]	2.0 [0.0–3.0]	0.035
Carbonated mineral water	1.0 [0.0–4.0]	1.0 [0.0–3.0]	0.549
Non-carbonated mineral water	5.0 [3.0–6.0]	7.0 [5.0–8.0]	0.025
Fruit juices	3.0 [2.0–4.0]	3.0 [2.0–3.0]	0.077
Vegetable juices	1.0 [0.0–3.0]	1.0 [0.0–2.0]	0.426
Fruit and vegetable juice	1.0 [0.0–3.0]	1.0 [0.0–3.0]	0.592
Non-carbonated fruit drinks	2.0 [0.0–3.0]	1.0 [0.0–2.0]	0.042
Sweetened carbonated drinks	2.0 [0.0–3.0]	1.0 [0.0–2.0]	0.046
Tea drinks	2.0 [0.0–3.0]	1.0 [0.0–2.0]	0.026
Cola drinks	2.0 [1.0–3.0]	2.0 [0.0–2.0]	0.801
Energy drinks	2.0 [0.0–3.0]	1.0 [0.0–1.0]	0.013
Isotonic drinks	1.0 [0.0–2.0]	1.0 [0.0–1.0]	0.204
Non-alcoholic beer	1.0 [0.0–2.0]	1.0 [0.0–2.0]	0.885
Beer	3.0 [1.0–4.0]	1.0 [1.0–3.0]	0.017
Wine	2.0 [1.0–2.0]	2.0 [1.0–2.0]	0.903
Vodka	2.0 [1.0–2.0]	1.0 [0.0–2.0]	0.006
Alcoholic drinks	1.0 [0.0–2.0]	1.0 [0.0–2.0]	0.894

HIS, Health Index Score; Q, quartile.

### 3.3. Anthropometric Measurements, Biochemical Analysis, and HIS Groups

A comparison of various health measurements, including blood pressure, anthropometric measurements, body composition (assessed by BIA), and biochemical analysis in urine, between the HIS groups is presented in Table 3. Many variables showed statistically significant differences between the two HIS groups, indicating associations between HIS levels and physiological and body composition parameters. The results indicate that individuals in the 0–2 HIS group had significantly higher SBP, DBP, BMI, and WC and less favorable body composition, characterized by a higher percentage of fat mass (FM) and a lower percentage of muscle mass (MM), fat-free mass (FFM), and TBW, despite potentially having higher absolute amounts (kg) of these components (likely due to overall larger body size) compared to the participants in the 3–5 HIS group. Additionally, this group had a higher amount of ECW and ICW compared to the 3–5 HIS group. Significant differences were noticed in the biochemical parameters of urine, with more concentrated urine (higher USG and Uosm) in the 0–2 HIS group compared to the 3–5 HIS group.

**Table 3:** Blood pressure, anthropometric measurements, body composition, and biochemical analysis in urine by HIS groups.

Variables	0–2 HIS (*n* = 115)	3–5 HIS (*n* = 222)	*p*-Value
	Median [Q1–Q3]		
Blood pressure			
SBP (mmHg)	125 [114.0–134.0]	118 [109.0–128.0]	0.001
DBP (mmHg)	79.0 [75.0–79.0]	77.0 [71.0–78.0]	0.002
Anthropometric measurements			
BMI (kg/m^2^)	25.1 [22.5–26.9]	21.8 [20.0–23.3]	0.001
HC (cm)	103 [97.0–109.0]	97.7 [91.5–100.0]	0.001
WC (cm)	82.0 [74.4–88.0]	72.0 [67.0–77.0]	0.001
HGS (kg)	38.0 [34.0–72.0]	37.0 [32.0–65.0]	0.736
Body composition—BIA			
MM (kg)	23.5 [20.0–31.2]	21.4 [19.3–25.4]	0.001
MM (%)	9.45 [9.0–15.7]	35.5 [13.0–21.5]	0.003
FFM (kg)	51.0 [45.1–65.9]	47.1 [42.9–56.2]	0.001
FFM (%)	75.7 [66.5–81.1]	78.0 [73.6–83.3]	0.002
TBW (kg)	38.4 [33.5–47.9]	34.2 [30.7–40.9]	0.001
TBW (%)	54.9 [50.2–58.8]	56.8 [52.7–60.3]	0.002
ECW (kg)	17.2 [15.5–19.9]	15.80 [14.1–17.7]	0.001
ECW (%)	45.1 [42.5–46.3]	44.3 [43.2–45.9]	0.321
ICW (kg)	20.2 [18.2–25.9]	18.9 [17.5–22.3]	0.001
ICW (%)	54.9 [53.7–57.5]	55.6 [54.0–56.8]	0.322
FM (kg)	17.6 [13.3–24.4]	13.5 [9.9–17.3]	0.001
FM (%)	24.3 [18.0–33.5]	21.9 [16.7–26.4]	0.005
Biochemical analysis—urine			
USG (g/mL)	1.024 [1.016–1.025]	1.019 [1.011–1.020]	0.038
Uosm (mOsm/kg)	572 [416.0–650.0]	516 [390.0–550]	0.027
pH	6.00 [5.9–6.5]	6.00 [5.9–6.5]	0.821

HIS, Health Index Score; Q, quartile; SBP, systolic blood pressure; DBP, diastolic blood pressure; BMI, body mass index; HC, hip circumference; WC, waist circumference; HGS, hand grip strength; MM, muscle mass; FFM, free-fat mass; TBW, total body water; ECW, extracellular water; ICW, intracellular water; FM, fat mass; USG, urine specific gravity; Uosm, urine osmolality; and pH, potential of hydrogen value.

In Table 4, the values of the HIS showed significant negative correlations with SBP (r = −0.139, *p* < 0.05) and DBP (r = −0.121, *p* < 0.05), which suggests that a higher level of general health is associated with lower blood pressure values. Moreover, strong negative correlations of HIS with BW (r = −0.411, *p* < 0.01), WC (r = −0.398, *p* < 0.01), and HC (r = −0.330, *p* < 0.01) clearly indicate that lower BW and smaller circumferences (especially around the waist, which is a marker of abdominal obesity) are closely associated with better HIS results. Positive correlation between HIS and TBW (r = 0.315, *p* < 0.01) and ICW (r = 0.139, *p* < 0.05) may suggest that optimal hydration and healthy body fluid composition are associated with better health indicators. On the other hand, extracellular water (ECW) showed a weak negative correlation with HIS (r = −0.130, *p* < 0.05).

**Table 4:** Partial Spearman correlation coefficients between the variables analyzed, adjusted for sex and age.

	HIS	SBP	DBP	BW	WC	HC	HGS	TBW	ICW	ECW	USG	Uosm	pH
HIS	1.000	−0.139 *	−0.121 *	−0.411 ***	−0.398 **	−0.330 **	−0.068	0.315 **	0.139 *	−0.130 *	−0.103 *	−0.085	−0.007
SBP	−0.134 *	1.000	0.549 ***	0.293 *	0.258 *	0.258 *	−0.015	−0.206 *	0.010	−0.001	0.084	0.094	−0.078
DBP	−0.121 *	0.546 ***	1.000	0.147 *	0.177 *	0.146 *	0.017	−0.180 *	−0.016	0.017	0.097	0.076	−0.147 *
BW	−0.411 **	0.299 *	0.147 *	1.000	0.817 ***	0.779 ***	0.116 *	−0.658 ***	−0.258 *	0.259 *	0.025	0.046	0.034
WC	−0.398 **	0.259 *	0.177 *	0.817 ***	1.000	0.654 ***	0.127 *	−0.714 ***	−0.202 *	0.203 *	0.019	0.037	0.010
HC	−0.330 **	0.258 *	0.146 *	0.779 ***	0.654 ***	1.000	0.038	−0.587 ***	−0.187 *	0.186 *	0.017	0.048	0.026
HGS	−0.068	−0.010	0.017	0.116 *	0.127*	0.038	1.000	−0.216 *	−0.104	0.100	−0.106	−0.097	−0.099
TBW	0.315 **	−0.200 *	−0.180 *	−0.658 ***	−0.714 ***	−0.587 ***	−0.216 *	1.000	0.099	−0.090	−0.040	−0.074	0.072
ICW	0.139 *	0.001	−0.016	−0.258 *	−0.202 *	−0.187 *	−0.104	0.099	1.000	−0.993 ***	−0.046	−0.068	0.050
ECW	−0.130 *	−0.001	0.017	0.259 *	0.203 *	0.186 *	0.100	−0.090	−0.993 ***	1.000	0.049	0.087	−0.057
USG	−0.103 *	0.083	0.097	0.025	0.019	0.017	−0.106	−0.040	−0.046	0.049	1.000	0.967 ***	−0.095
Uosm	−0.085	0.094	0.076	0.046	0.037	0.048	−0.097	−0.074	−0.068	0.087	0.967 ***	1.000	−0.160
pH	−0.007	−0.078	−0.147 *	0.034	0.010	0.026	−0.099	0.072	0.050	−0.057	−0.095	−0.160	1.000

HIS, Health Index Score; SBP, systolic blood pressure (mmHg); DBP, diastolic blood pressure (mmHg); BW, body weight (kg); WC, waist circumference (cm); HC, hip circumference (cm); HGS, handgrip strength of arm muscles (kg); TBW, total body water (%); ICW, intracellular water (%), ECW, extracellular water (%); USG, urine-specific gravity (g/mL); Uosm, urine osmolality (mOsm/kg); pH, potential hydrogen value; * *p* ≤ 0.05; ** *p* ≤ 0.01; and *** *p* ≤ 0.001.

The analysis of the data presented in Table 5 shows the predictive models for health status and hydration status in adults based on the 0–2 HIS group. Model 1 was adjusted for age, sex, and place of residence. In the health status domain, the significant predictors were fatigue during the day (OR: 1.60; 95% CI: 1.05–3.63), SBP (OR: 1.46; 95% CI: 1.02–1.68), WC (OR: 1.29; 95% CI: 1.07–1.43), and percentage of FM (OR: 2.01; 95% CI: 1.21–2.67). In relation to hydration status, the frequency of non-carbonated mineral water consumption (OR: 0.82; 95% CI: 0.11–0.94) and Uosm (OR: 1.61; 95% CI: 1.27–2.48) were significant predictors. Model 2 was adjusted for sex, age, place of residence, education, and economic status. The significant variables were fatigue during the day (OR: 1.45; 95% CI: 1.11–1.78), WC (OR: 1.35; 95% CI: 1.15–1.57) in health status, and consumption of non-carbonated mineral water (OR: 0.54; 95% CI: 0.21–0.86) and Uosm (OR: 1.87; 95% CI: 1.33–2.37) in hydration status. Although Table 2 showed multiple beverage differences by HIS, only non-carbonated mineral water remained an independent predictor in adjusted regression.

**Table 5:** Predictive model for health status and hydration status in adults based on HIS group.

Variables	0–2 HIS				
	Β ^a^	Eβ ^b^	95% CI ^c^		*p*-Value ^d^
Model 1					
Health status					
Fatigue during the day	0.470	1.60	1.05	3.63	0.039
SBP (mmHg)	0.378	1.46	1.02	1.68	0.042
WC (cm)	0.257	1.29	1.07	1.43	0.001
FM (%)	0.699	2.01	1.21	2.67	0.024
Hydration status					
Non-carbonated mineral water ^e^	−0.198	0.82	0.11	0.94	0.014
Uosm	0.475	1.61	1.27	2.48	0.017
Model 2					
Health status					
Fatigue during the day	0.580	1.45	1.11	1.78	0.025
WC (cm)	0.347	1.35	1.15	1.57	0.031
Hydration status					
Non-carbonated mineral water ^e^	−0.217	0.54	0.21	0.86	0.019
Uosm	0.511	1.87	1.33	2.37	0.024

^a^, estimate; ^b^, OR-point estimate (eβ); ^c^, 95% confidence intervals; ^d^, the Wald test; Model 1 adjusted for sex, age, and place of residence; Model 2 adjusted for sex, age, place of residence, education, and economic status; ^e^, the data presented relate to the frequency of consumption; HIS, Health Index Score; SBP, systolic blood pressure; WC, waist circumference; FM, fat mass; and Uosm, urine osmolality.

## 4. Discussion

The group under analysis contained 115 individuals (34%) in the 0–2 HIS group and 222 (66%) in the 3–5 HIS group. This finding shows that approximately 1/3 of the respondents exhibited signs of unfavorable health-related behaviors. Individuals in the 0–2 HIS group were more likely to be male, smoke, have low physical activity, be overweight or obese, sleep < 6 h, and/or consume alcohol ≥ 2 times/week. In contrast, a higher HIS (3–5) was associated with female gender, non-smoking, moderate-to-high physical activity, normal BMI, adequate sleep, and lower alcohol consumption. These findings suggest that HIS is strongly associated with modifiable lifestyle factors and may serve as a useful tool in health promotion strategies. The study established that fatigue during the day and a 1-unit increase in SBP, WC, FM (%), and Uosm were linked with unhealthy habits in the study group. In turn, an increased frequency of non-carbonated mineral water consumption was linked with a decreased likelihood of unhealthy habits.

### 4.1. The Relationship Between Sociodemographic Factors and Health-Related Behaviors

In the analyzed sample, gender was shown to be a significant factor in the frequency of healthy behaviors. Compared to men, women were more likely to report healthy habits, such as avoiding smoking, consuming alcohol in moderation, being physically active, and getting enough sleep. These results are consistent with previous studies, which have shown that women are more likely than men to take health-promoting actions and are also more willing to change their lifestyle to improve their health [31,32]. Conversely, men were more likely to engage in risky behaviors such as smoking, excessive alcohol consumption, and lower levels of physical activity. These trends may result from cultural conditions, gender stereotypes, and differences in the perception of health and risk. Studies also show that men are less likely to participate in preventive activities and are less willing to undertake health interventions [33]. These differences highlight the importance of adapting educational and preventive activities to gender-specific factors, which could lead to more effective health promotion among adults [32].

People with healthy habits are much less likely to smoke, which suggests that they are more aware of and concerned about their health. Not smoking is a fundamental element of a healthy lifestyle and correlates with other beneficial habits [34]. Regular physical activity is another important component of healthy behavior—physically active people are more likely to be concerned about their health, which translates into avoiding smoking and other harmful habits [35]. A healthy BMI is often the result of a balanced diet and exercise alongside other healthy habits, such as not smoking and moderate alcohol consumption [36]. Equally important is getting the right amount of sleep, as people who care about their health pay attention to bodily regeneration and avoid sleep disorders that can lead to risky behaviors such as smoking or excessive drinking [37]. Finally, low or occasional alcohol consumption is characteristic of people with a healthy lifestyle [38]. They combine this habit with physical activity, proper nutrition, and not smoking. These patterns suggest that clusters of healthy behaviors reinforce each other, supporting long-term health maintenance.

Recent studies further confirm this association. For instance, findings from the Healthy Finland Survey demonstrated that the prevalence of metabolic syndrome was significantly lower in individuals with a normal BMI and the healthiest lifestyle, compared to those with obesity and the least healthy behaviors. Among men, the risk of metabolic syndrome decreased across BMI categories as lifestyle scores improved, suggesting that healthy lifestyle behaviors can attenuate the metabolic risk associated with higher body weight [39]. Similar patterns were observed among dental students, where obesity was significantly associated with unhealthy dietary habits, physical inactivity, and poor sleep hygiene. More than half of the obese participants reported no physical activity and insufficient sleep, highlighting the strong link between lifestyle and BMI in younger adults as well [40]. Moreover, research among gym-goers has shown that individuals who regularly engage in physical activity and follow balanced diets are more likely to maintain a healthy BMI, reinforcing the idea that exercise and proper nutrition are essential for positive health outcomes and long-term lifestyle maintenance [41].

### 4.2. Health Behaviors and Health Status and Hydration Status

Unhealthy habits among Polish adults were found to be linked with daytime fatigue in our study. The results of the study suggest that feeling tired during the day may be significantly linked to unhealthy habits. People who reported frequent tiredness were more likely to report shorter than recommended sleep time, a lack of regular physical activity, and an incorrect BMI [42,43]. Daytime tiredness may also be a consequence of poor sleep hygiene, often accompanied by behaviors such as excessive use of electronic devices before sleep, smoking, or consuming alcohol in the evening, all of which negatively affect sleep quality and the body’s ability to regenerate [44,45]. The literature emphasizes that such unhealthy habits as a sedentary lifestyle, an improper diet, and stimulant use can lead to chronic fatigue, reduced energy levels during the day, and impaired cognitive function [42,44]. Conversely, fatigue can perpetuate unhealthy behaviors: chronically tired individuals are less likely to engage in physical activity and more likely to opt for quick, high-calorie meals and psychoactive substances, creating a vicious cycle that exacerbates health issues [46,47].

It was found by the present study that unhealthy habits were linked with a 1-unit increase in SBP. Participants with higher SBP and DBP values are more likely to exhibit the following behaviors: a lack of regular physical activity; abnormal body weight (being overweight or obese); poor diet (consuming high levels of salt and highly processed foods); poor sleep quality; and excessive alcohol consumption. These factors are widely recognized as major modifiable lifestyle risk factors for developing hypertension [48]. Physical inactivity weakens blood vessels, increasing vascular resistance and promoting chronic hypertension. Additionally, excess body weight, particularly abdominal obesity, is associated with increased activity of the sympathetic nervous system and the renin–angiotensin–aldosterone system, contributing to increased blood pressure. Inadequate sleep quantity and poor sleep quality can affect blood pressure by disrupting circadian rhythms, increasing cortisol levels and activating the hypothalamic–pituitary–adrenal axis [49]. Furthermore, regular alcohol consumption exceeding recommended levels leads to a gradual increase in blood pressure, and the simultaneous occurrence of several of these habits can amplify their harmful effect [50]. Therefore, hypertension should be considered in the context of the cumulative impact of many unhealthy behaviors, and modifying these could significantly improve population health outcomes.

This study demonstrated a significant association between a 1-unit increase in WC and FM (%) and unhealthy habits in the study group. An increased waist circumference and higher body fat content are significantly associated with unhealthy habits. Population studies have shown that a lack of regular physical activity, an excessive calorie intake (particularly from highly processed foods), poor sleep quality, smoking, and consuming alcohol in amounts that exceed recommendations are the main factors that contribute to the accumulation of body fat, particularly visceral fat [51,52,53,54]. A high waist circumference is a key indicator of abdominal obesity, which increases the risk of developing insulin resistance, type 2 diabetes, hypertension, and cardiovascular disease [55]. Incorrect eating habits, such as irregular mealtimes, snacking, and consuming excess saturated fat and simple sugars, are directly related to the accumulation of visceral fat [56,57]. People with low levels of physical activity not only have a higher overall level of body fat, but also have an unfavorable distribution of it—abdominal obesity [58]. Insufficient sleep disrupts the hormonal regulation of appetite (leptin and ghrelin), leading to overeating and increased fat mass. Alcohol consumption, particularly of beer and spirits, is also associated with a positive energy balance and increased waist circumference, even in people with an apparently normal BMI [59].

We demonstrated that a 1-unit increase in Uosm was associated with unhealthy habits among Polish adults. The results indicate that increased urine osmolality, a sign of hypohydration, is significantly associated with several adverse health behaviors. Firstly, inadequate hydration (osmolality > 800 mOsm/kg) frequently occurs alongside low levels of physical activity—less active individuals are less likely to replenish fluids, resulting in a high urine concentration [60,61]. Furthermore, individuals with a higher BMI and larger waist circumference have a greater need for water and exhibit higher osmolality values, potentially due to lower hydration awareness and dietary habits that promote dehydration [62]. Smoking and alcohol consumption, particularly in the evening, also contribute to dehydration: alcohol has a diuretic effect and smoking worsens sleep quality, limiting regular fluid intake. Clinical studies have shown that people who consume large quantities of alcohol have higher urine osmolality and more concentrated excretions [63]. Furthermore, reduced sleep duration correlates with increased osmolality as disruption to nocturnal rhythms limits fluid intake and promotes morning dehydration [64]. Taken together, these data support the conclusion that increased urine osmolality reflects not only inadequate hydration, but also a general pattern of unhealthy behaviors, including low physical activity, being overweight, poor sleep, stimulant use, and a lack of health literacy.

The directionality of the observed associations appears to be clinically meaningful—data from a nationally representative NHANES sample indicate that underhydration (e.g., urine osmolality ≥ 500 mmol/kg) is associated with higher risks of obesity, insulin resistance, type 2 diabetes, metabolic syndrome, and increased chronic disease mortality. Among underhydrated individuals, the risk of death from chronic diseases was over four times higher (HR = 4.21; 95% CI: 1.29–13.78; *p* = 0.019) compared to those meeting hydration criteria. Notably, no chronic disease deaths were observed among individuals who were adequately hydrated and free from chronic conditions at baseline. These findings highlight the potential clinical relevance of hydration markers in the context of chronic disease prevention and public health [65].

Further supporting this, Jacques et al. [66] demonstrated that higher water intake and more favorable hydration markers were associated with improved lipid metabolism and reduced cardiometabolic risk in older adults, suggesting that hydration plays a significant role in maintaining metabolic health. Additionally, recent longitudinal findings from the large-scale cohort in China, involving over 71,000 participants followed for more than 12 years, revealed that suboptimal hydration status—measured by urine specific gravity—was significantly associated with an increased risk of developing type 2 diabetes [67]. Compared to individuals with optimal hydration, those with dehydration and severe dehydration had a 30% and 38% higher risk of developing T2D, respectively. Time-dependent analyses further showed that even impending dehydration increased diabetes risk by 16%, emphasizing the importance of adequate hydration in the early prevention of T2D.

Ultimately, accumulating evidence indicates that inadequate hydration is not only common but also clinically significant, as it may contribute to elevated risks of metabolic disorders and premature mortality. These findings reinforce the importance of monitoring and maintaining proper hydration as a modifiable factor in chronic disease prevention strategies.

Our study showed that an increased frequency of non-carbonated mineral water consumption was linked with a decreased likelihood of unhealthy habits. An increased frequency of drinking still water is strongly associated with an improved quality of life and a reduced risk of unhealthy behaviors. An analysis of data from the 2017 National Youth Risk Behavior Surveillance System found that American adolescents who drank water three or more times per day were more likely to report regular physical activity and higher fruit and vegetable consumption. They were also less likely to consume sweetened beverages [68]. Similarly, a study of Chinese young adults found that individuals who were adequately hydrated—those who drank mineral or tap water regularly—had lower urine osmolality and consumed significantly fewer sugary beverages. They were also more likely to meet the criteria for a healthy dietary profile [63]. In addition, research using data from NHANES on adults with NAFLD/MASLD showed that switching from sweetened beverages to plain water was linked to a lower mortality rate. This could be because there was less dehydration and fewer metabolic issues [69].

### 4.3. Strength and Limitations of the Study

A major strength of this study is the inclusion of participants from varied social backgrounds—encompassing both smaller and larger towns, a range of economic statuses, and differing levels of education—which allowed for a more representative reflection of the diversity within Polish society. Additionally, the data collection was carried out through direct, face-to-face contact rather than online, which facilitated the clarification of any doubts respondents had while completing the questionnaire. This likely improved both the accuracy and completeness of the responses. Another strength lies in the objective nature of several measurements. BP, anthropometric parameters, and body composition analyses were performed by trained personnel, while urine biochemical assessments were carried out in a certified diagnostic laboratory. The study also employed a multimodal approach to assess health status, incorporating physical activity, BMI, smoking status, alcohol consumption, and sleep status. Moreover, additional indicators—including TBW, USG, and Uosm—were measured to ensure a comprehensive evaluation of hydration status.

Despite these strengths, certain limitations should be acknowledged. First, although the sample size was in line with the assumptions of statistical calculation, it remains relatively small, and the obtained sample, despite its diversity, may not fully reflect the structure of the entire Polish population. In the future, it would be advisable to compare the characteristics of the sample with national data in order to increase the representativeness of the results. Next, the study did not include an assessment of food consumption, which prevented the analysis of food products that were a source of water. Thirdly, self-reported measures used in this study, including the FFQ, as well as data on sleep, smoking, and alcohol consumption, may be subject to reporting bias (such as retrospective memory or underestimation), which should be taken into account when interpreting the findings. However, it should be emphasized that the FFQ is a recognized, verified, and commonly used tool in epidemiological studies. Additionally, it is worth noting that the analysis of the first morning urine sample may be subject to slight error, as studies show that this sample has the highest osmolality. Nevertheless, this is a widely accepted procedure in scientific hydration research [70,71].

The study revealed various potential correlations between the examined factors. While these associations are noteworthy, several interpretations—such as the suggestion that dehydration may indirectly affect general well-being by increasing fatigue and leading to less favorable health decisions—should be considered speculative and treated as hypotheses for future research, given the lack of direct evidence in the current dataset. Moreover, due to the cross-sectional design of the study, the directionality of observed relationships cannot be determined—it remains unclear whether dehydration contributes to fatigue and poor health decisions, or whether individuals with such behaviors are less likely to hydrate properly.

## 5. Conclusions

The results obtained indicate a strong association between the HIS and modifiable lifestyle factors. This suggests its potential usefulness as a simple tool for supporting health promotion and non-communicable disease prevention activities. Additionally, daily fatigue, increased SBP, WC, FM (%), and Uosm were found to be significantly associated with unhealthy habits. Conversely, more frequent consumption of still mineral water was associated with a reduced risk of adverse health behaviors. The results of the study also showed that hydration may play an important role in promoting healthy behaviors. Increased Uosm, which indicates possible dehydration, was significantly associated with unhealthy habits. Conversely, more frequent consumption of still mineral water was associated with a lower risk of adverse health behaviors, emphasizing the importance of adequate hydration in health prevention. In summary, the HIS index and hydration-related parameters can complement the assessment of the health status of the adult population and the identification of groups requiring special support in health promotion interventions.

Importantly, the findings of this study offer practical value for public health practice: the HIS index can serve as an accessible, non-invasive screening tool to identify individuals at higher risk of unhealthy behaviors and even early metabolic alterations. Given its simplicity, it may be effectively implemented in workplace health programs, primary care settings, or community-based health campaigns. Moreover, the observed associations between hydration status and health-related behaviors highlight a frequently overlooked but modifiable factor that can be targeted to improve overall lifestyle quality. These insights can inform the design of more tailored and efficient health promotion strategies, particularly for adult populations at risk of non-communicable diseases.

## Data Availability

The raw data supporting the conclusions of this article will be made available by the authors on request due to to legal and ethical restrictions concerning confidentiality and personal data protection.

## Abbreviations

The following abbreviations are used in this manuscript:

BIA	Bioelectrical Impedance Analysis
BMI	Body Mass Index
BP	Blood Pressure
BW	Body Weight
CI	Confidence Intervals
DBP	Diastolic Blood Pressure
FFM	Fat-free Mass
FFQ	Food Frequency Questionnaire
FM	Fat Mass
H	Height
HC	Hip Circumference
HGS	Handgrip Strength
HIS	Health Index Score
IPAQ	International Physical Activity Questionnaire
MET	Metabolic Equivalent of Work
MM	Muscle Mass
OR	Odds Ratio
Q	Quartile
pH	Potential Hydrogen Value
SBP	Systolic Blood Pressure
TBW	Total Body Water
Uosm	Urine Osmolality
USG	Urine Specific Gravity
WC	Waist Circumference
WHO	World Health Organization
